# Comparison of endoscopic injection of botulinum toxin and steroids immediately after endoscopic submucosal dissection to prevent esophageal stricture: a prospective cohort study

**DOI:** 10.7150/jca.60720

**Published:** 2021-08-02

**Authors:** Xiaoying Zhou, Han Chen, Meihong Chen, Chao Ding, Guoxin Zhang, Xinmin Si

**Affiliations:** Department of Gastroenterology, Jiangsu Province Hospital and The First Affiliated Hospital of Nanjing Medical University, Nanjing, Jiangsu, China.

**Keywords:** endoscopic submucosal dissection, esophageal stricture, endoscopic injection, botulinum toxin type A, triamcinolone acetonide, prospective cohort study

## Abstract

**Background:** Widespread endoscopic submucosal dissection (ESD) in early esophageal cancer patients is closely associated with esophageal stricture, which dramatically reduces patients' quality of life and increases huge medical burdens. Endoscopic injection of steroid was proved as a protective method for post-ESD strictures. Other materials such as botulinum toxin type A (BTX-A) may be potential candidates. We conducted this prospective cohort study to compare the efficacy and feasibility of endoscopic injection of BTX-A and triamcinolone acetonide (TA) for the prevention of esophageal stricture.

**Methods:** Seventy-eight patients with esophageal mucosal defects of more than two thirds of the circumference were successively enrolled and divided into 3 groups: BTX-A group (group A, n=26), TA group (group B, n=16) and control group (group C, n=36). Patients in group A were immediately injected with BTX-A after ESD, in group B were immediately injected with TA and in group C received ESD only. Endoscopy was performed when patients reported dysphagia symptoms and at 6 and 12 weeks post-ESD in patients without symptoms. Patients who experienced post-ESD esophageal strictures in all groups received bougie dilation. All patients were followed up for one year.

**Results:** The proportion of patients developing stricture in BTX-A group was 30.00% (intention to treat analysis, 9/30) and 26.92% (per protocol analysis, 7/26), in TA group was 40.90% (intention to treat analysis, 9/22) and 43.75% (per protocol analysis, 7/16), and in control group was 84.21% (intention to treat analysis, 32/38) and 83.33% (per protocol analysis, 30/36) (p<0.001). When further comparing between each of the two groups, the incidence of esophageal stricture was lower in BTX-A group than that in control group (p<0.001), and lower in TA group than that in control group (p=0.004). Furthermore, in entire circumference mucosal defect subgroup, the esophageal stricture was significantly lower in BTX-A group than that in TA group (33.3% vs 100%, p=0.0454).

**Conclusions:** Endoscopic injection of BTX-A and TA were effective in preventing post-ESD esophageal strictures and BTX-A injection was particularly effective in entire circumference mucosal defect patients. Multi-centered, randomized prospective study with larger sample size should be conducted. (Clinical trial registration number: ChiCTR2100042970, registered 1 February 2021, retrospectively registered, http://www.chictr.org.cn/listbycreater.aspx)

## Background

Endoscopic resection is globally accepted as a minimally invasive treatment for early esophageal cancer 1. As an alternative to esophagectomy, endoscopic mucosal resection (EMR) had been originally applied for esophageal squamous cell carcinoma in 1990s 2. Recently, EMR has been gradually replaced by endoscopic submucosal dissection (ESD), as ESD allows the entire resection of the lesion regardless of its size and has a lower recurrence rate compared to EMR 3. However, the residual mucosal defect after ESD may cause acute inflammation, deep ulcers, local submucosal fibrous connective tissue proliferation, collagen deposition, esophageal wall fibrosis, and esophageal stricture formation 4, 5.

The incidence of esophageal strictures after endoscopic resection resulting in large near-circumferential or circumferential esophageal mucosal defects has been reported to be 88% to 100% 6. Although endoscopic balloon dilation is effective for the treatment of strictures, it is performed repeatedly until dysphagia resolves 7. Repeated dilation not only increases the risk of perforation and bleeding and the burden of society but also reduces the patient's quality of life 8. A review report investigating 73 studies suggested that oral triamcinolone acetonide (TA) administration, not prophylactic endoscopic balloon dilation alone, was effective in preventing esophagostenosis and reducing the number of repeated endoscopic balloon dilations even after extensive endoscopic resection 9. Local steroid injection is useful and economy for preventing esophageal stricture, even though it may raise the risk of perforation during dilations 10. Jing et al concluded for the first time that a single injection of botulinum toxin type A (BTX-A) reduced esophageal stricture rate and the times of bougie dilation procedures, as BTX-A was reported to be used to decrease the fibrosis of a surgical wound and prevent widening of facial scars 11. BTX-A can also down-regulate the expression of both transforming growth factor-b1 mRNA and transforming growth factor-b1 protein in the esophageal scar tissues, leading to less deposition of both type I and type III collagen in the tissues 12.

A preliminary aim of this study was to determine the relationship between the extent of the esophageal mucosal defect after ESD and the risk of stricture formation. Specifically, we determined the risk of strictures in ESD patients with mucosal defects more than one half of the circumference of the esophagus after ESD treatment. Our prospective study's primary aim was to investigate the efficacy and feasibility of the endoscopic injection of BTX-A and TA for the prevention of esophageal strictures after ESD for early esophageal cancer.

## Methods

### Study patients

Originally, 80 patients with early esophageal cancer who underwent ESD at First Affiliated Hospital of Nanjing Medical University from March 2018 to May 2019 were randomly divided into BTX-A group (group A), TA group (group B) and control group (group C). The inclusion criteria for the study were as follows: 1) preoperative pathology indicating precancerous lesions or carcinoma in situ, 2) mucosal defects exceeding two thirds of the circumference of the esophagus, 3) the absence of lymph node metastases confirmed by CT, 4) no contraindications to general intravenous anesthesia, 5) no serious cardiopulmonary disease, 6) the patient's signed informed consent. However, we excluded patients who received additional therapy such as chemotherapy, radiotherapy or additional surgery and those who had non-curative ESD procedures. Patients who diagnosed with invasive esophageal carcinoma or tumor recurrence were also excluded in our study. Patients suffering from other diseases of esophagus such as congenital anatomical structure abnormality, esophageal varices, functional esophageal disease, severe reflux esophagitis were also excluded from the study.

Our study has been approved by the Ethics Committee of First Affiliated Hospital of Nanjing Medical University and written informed consent was obtained from each patient. The ethical approval number for this study was 2018-SR-199. (Clinical trial registration number: ChiCTR2100042970).

### Study design

We designed a prospective cohort study. Before ESD procedure, all patients were prospectively divided into three groups. Patients in group A (BTX-A group, n=30) received endoscopic BTX-A injection immediately after ESD procedure, patients in group B (TA group, n=20) received TX injection immediately after ESD, and patients in group C (control group, n=40) received ESD only without subsequent injection. The mucosal defect after ESD was classified into three groups based on the extent of the areas affected: two thirds to three fourths of the esophageal circumference, three fourths to the full esophageal circumference and full circumferential mucosal defect. In either group A, B or C, all patients with full circumferential mucosal defects received oral prednisolone after ESD.

### ESD procedures

Endoscopic procedures were carried out with an upper endoscope with an outer diameter of 9.9 mm (GIF-Q260J; Olympus Medical System Co., Tokyo, Japan). The electrosurgical unit and knife for ESD consisted of a high frequency generator (VIO300; ERBE Elektromedizin, Tubingen, Germany), the HookKnife and the DualKnife (Olympus Co.). Submucosal dissection was carried out with the autocut mode (60 W, effect, 5) to decrease the burning effect on the resected surface, which could reportedly cause severe stenosis after extensive esophageal ESD. The coagulation mode was used only to stop bleeding and for preventive vascular coagulation.

### Endoscopic BTX-A and TA Injection procedure

Just after dissection and hemostasis, a single-session endoscopic BTX-A or TA injection was administered. BTX-A solution (Lanzhou Institute of Biological Products, Lanzhou, China) was injected in 5-mL increments into 10 separate points at the level of the muscularis propria equally spaced along the circumference of the defect with a 25-gauge, 4-mm needle (TOP Corporation, Tokyo, Japan). TA was diluted with 0.9% NaCl to a final concentration of 4mg/ml. A total of 40 mg (10ml) TA was injected into the deep submucosa of the ulcer base at 10 sites, with a 1 mL dose at each site. A total of 100 units of BTX-A was diluted with 5 mL of saline solution (20 units/mL). Where BTX-A or TA, the injections were placed along the junction of the defect and the normal tissue. However, patients with full circumferential mucosal defects were injected superficially into the base of the cautery ulcer. All patients received the same dose of BTX-A (100 units) and TA (40mg), regardless of the lesion size.

### Postoperative management and follow-up

All patients were requested fasting the first day after ESD, and liquid diets for the next several days. Proton pump inhibitor, antibiotic, and hemostatic were routinely used to promote rehabilitation. The occurrence of perforation, hemorrhage, fever, chest pain, allergy and other adverse events are paid closely attention. In our study, 6 patients had small amount of bleeding during the ESD procedure and all resolved by endoscopic hemostasis with hot forceps. No delay bleeding was occurred in these patients. 24 patients had muscular injury during the ESD procedure, which means myometrial exposure of the wound. The wounds were clipped by titanium clips and no patients were suffered with perforation. Patients with entire circumferential ESD were administered a systemic steroid. Oral prednisolone was started at a dose of 30 mg/day on the second day post-ESD, tapered gradually (30, 30, 30, 30, 25, 25, 20, 20, 17.5, 17.5, 15, 15, 12.5, 10, 7.5 and 5 mg for 7 days each) and then discontinued 16 weeks (112 days) later. Follow-up endoscopy was scheduled at 6 and 12 weeks after ESD, and telephone follow-up was conducted every week post-ESD for dysphagia and Quality of Life Questionnaire (EORTC QLQ-OES18) scores. Dysphagia was evaluated using the Mellow-Pinkas score as follows: 0=no dysphagia, 1=dysphagia to normal solids, 2=dysphagia to soft solids, 3= dysphagia to solids and liquids, and 4=complete dysphagia, even to saliva. Bougie dilation using Savary-Gilliard dilators (Wilson-Cook Medical, Winston-Salem, NC) was applied as needed whenever the patients complained of dysphagia. In cases of persistent dysphagia, bougie dilation was performed until dysphagia resolved.

### Endpoints

Esophageal stricture is defined as a symptomatic dysphagia and/or impossible passage of a standard endoscope at the stricture. Through endoscopy and telephone follow-up, we recorded the number of patients with stricture in each group, and the number of required dilatation procedures for treatment of a stricture, the dysphagia grading score, the Quality of Life Questionnaire (EORTC QLQ-OES18) score, the asymptomatic remission period, the diameter of a narrow esophagus, and the time of first stenosis occurred. During the asymptomatic remission period, patients had a dysphagia score larger than 2, and did not need endoscopic dilatation. The primary endpoint of our study was the stricture rate after ESD with or without BTX-A/TA injection. Secondary endpoints were the number of dilatation procedures, the dysphagia and Quality of Life Questionnaire (EORTC QLQ-OES18) scores, the asymptomatic remission period, the diameter of a narrow esophagus, and the time of first stenosis occurred. Since this study was a prospective cohort study, we defined TA and BTX-A injection as different exposure factors, and the endpoints we mentioned above were the indicators we compared.

### Statistical analysis

PASS 15 (Version: 15.0.13) was used for determining sample size in this study. Section of Proportions in PASS for Chi-Square (Contingency Table/Crosstabs) Tests was applied for sample calculation. Based on the 3*2 Contingency Table, Degrees of Freedom was 2 with a power of 0.90, Alpha of 0.05, and W (Effect Size) of 0.432049 (calculated by typing an expected stricture rate of 20% in BTX, 40% in TA and 80% in control groups, respectively). We calculated a sample size of at least 68 patients required for statistical analysis. Based on this, we finally enrolled 90 patients when considering an approximately 20% drop-up rate.

Normality tests were applied by Shapiro-Wilk and Kolmogorov-Smirnov test. To compare the means from three groups, One-Way ANOVA was applied in continuous variables with normal distribution and Kruskal-Wall H-test for those with the abnormal distribution. Chi-Square Test or Fisher's Exact Test were used for in dichotomous variables (3×2 Contingency Tables) to compare treatment effects among three groups. Pairwise comparisons were conducted in Contingency Tables using an adjusted α level after Bonferroni correction method. Logistic regression was performed to identify any significant risk factors of esophageal stricture. Statistical analysis was performed with IBM SPSS Statistics for Windows, Version 23.0 (SPSS, Chicago, IL). P values < 0.05 were considered statistically significant in general comparisons among three groups. For pairwise comparisons, P values < 0.0167 (0.05/3) were considered statistically significant after Bonferroni correction test.

## Results

### Patient characteristics

**Figure 1:**
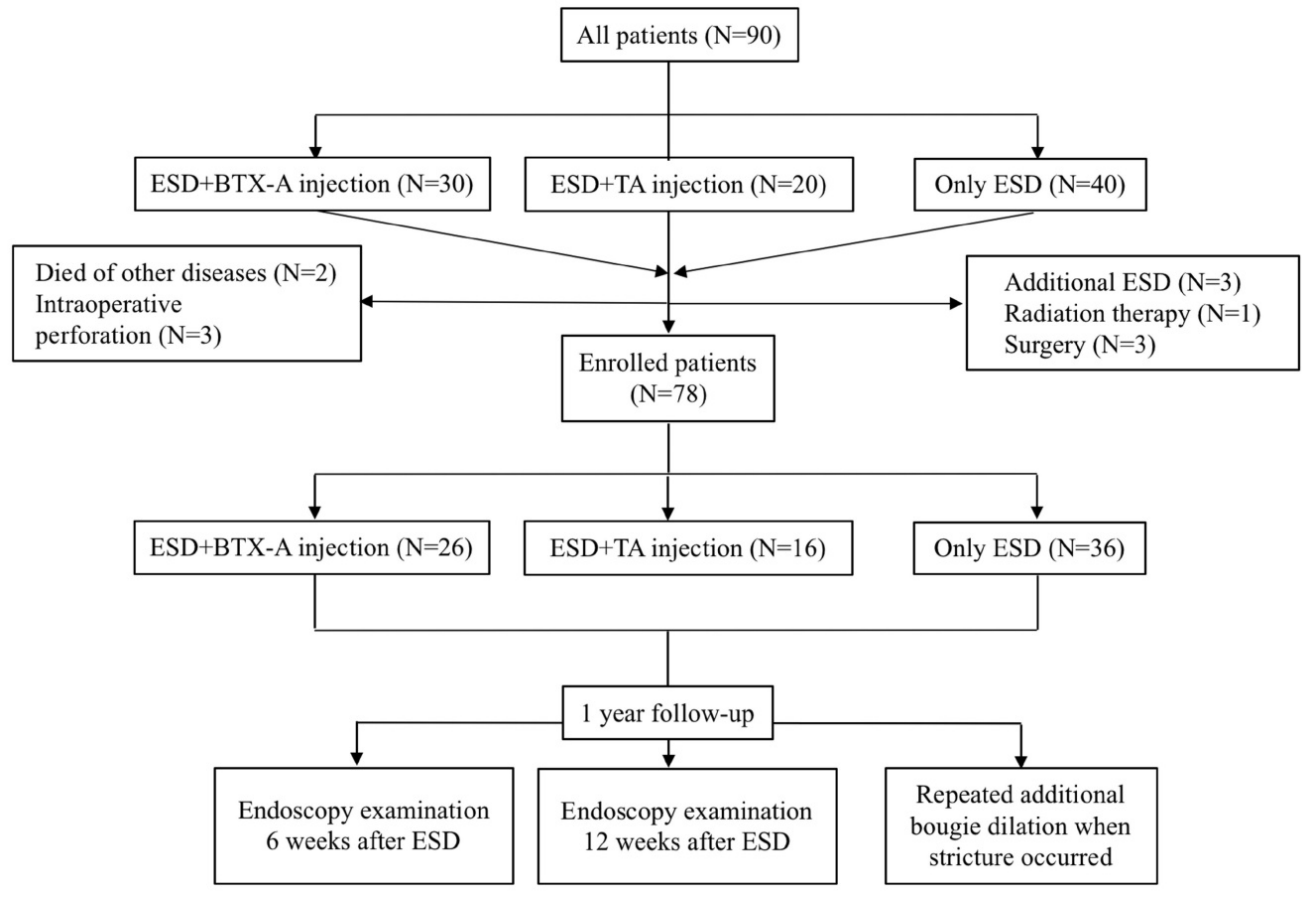
Flow diagram showing inclusion and exclusion of patients for the three study groups. ESD, endoscopic submucosal dissection; BTX-A, botulinum toxin type A; TA, triamcinolone acetonide.

Ninety patients who fulfilled the inclusion and exclusion criteria with an esophageal defect greater than two thirds of the circumference were enrolled in this study, of whom 30 received BTX-A injection immediately after ESD procedure, 20 received TA injection immediately after ESD procedure and 40 only received ESD. Seven patients were excluded from the study because they received additional treatments such as additional ESD (N=3), surgery (N=3) and radiation therapy for the non-curative resection based on the postoperative pathologic diagnosis (N=1). Two patients died of non-digestive diseases and three patients had intraoperative perforation and were also excluded from our study (Figure 1). We compared the efficacy of BTX-A injections, TA injections and controls for esophageal stricture from the remaining 78 patients. Baseline data of the patients and treatment outcomes were summarized in Table 1. There was no significant difference between the 3 groups with respect to age, sex, hospital stay days, BMI, smoking history, drinking history, family history of esophageal tumors, location of the lesion, post-operative pathology, circumferential range, longitudinal resection length, depth of infiltration, rate of en-bloc resection, operating time for ESD and adverse events such as muscular injury and hemorrhage.

### Primary outcome

As shown in Table 2, we found that the proportion of patients developing stricture in BTX-A group was 30.00% (intention to treat analysis, 9/30) and 26.92% (per protocol analysis, 7/26), in TA group was 40.90% (intention to treat analysis, 9/22) and 43.75% (per protocol analysis, 7/16), and in control group was 84.21% (intention to treat analysis, 32/38) and 83.33% (per protocol analysis, 30/36) (p<0.001). When further comparing between each of the two groups, the incidence of esophageal stricture was lower in BTX-A group than that in control group (p<0.001, χ2=19.964, OR=0.074 (0.021-0.253)), and lower in TA group than that in control group (p=0.004, χ2=8.456, OR=0.156 (0.042-0.583)). However, the incidence of esophageal stricture was found no significantly difference between BTX-A and TA group (p=0.261, χ^2^=1.262, OR=2.111 (0.567-7.855)) (Table 3).

Subgroup analysis according to the extent of the defect after ESD was further conducted to compare the esophageal stricture rates among the three groups (Table 4). No patient in BTX-A and TA groups in the two thirds to three fourths circumference mucosal defect subgroup developed esophageal stricture. Five patients developed stricture in the two thirds to three fourths circumference mucosal defect subgroup in the control group. In the three fourths to nearly full circumference mucosal defect subgroup, 45.5% (5/11) patients from group A, 37.5% (3/8) patients from group B and 88.2% (15/17) of group C patients developed strictures. In patients with entire circumference mucosal defect subgroup, 33.3% (3/6) of group A, 100% (4/4) of group B and 60% (10/10) of group C patients developed strictures. Patients with full circumferential mucosal defects received additional oral prednisolone administration. Although the results of esophageal stricture rate showed that no significant difference was found in BTX-A and TA group, in entire circumference mucosal defect subgroup, the esophageal stricture was significantly lower in BTX-A group than that in TA group (33.3% vs 100%, p=0.0454).

### Secondary outcome

After ESD procedures, patients in group A required an average of 1.19 bougie dilations (range, 0-12), patients in group B required an average of 1.31 bougie dilations (range, 0-9), whereas patients in group C required an average of 3.14 bougie dilations (range, 0-16) (p=0.019). The mean Quality of Life Questionnaire (EORTC QLQ-OES18) score in group A was 24.15±2.19, in group B was 24.88±2.13 and in group C was 23.47±1.1 (p=0.029). The grading of dysphagia was 0.5 (0-3), 1.5 (0-3) and 2 (0-4) in group A, B and C, respectively (p=0.009). As shown in Table 5, there was a significant difference among the three groups in the Atkinson rating of dysphagia, the Quality of Life Questionnaire (EORTCQLQ-OES18) scores, the number of bougies needed after ESD, asymptomatic remission periods and diameter of narrow esophagus (P < 0.05) (Table 5). Two representative case in the group A and B are shown in Supplementary Figure 1 and 2.

Logistic regression analysis was conducted. The following factors of sex, age, smoking and drinking history, family history of esophageal tumor, location of lesion, longitudinal resection length, post-operation pathology and oral prednisolone were not relevant to the development of postoperative esophageal strictures. However, circumferential range (OR: 38.600; 95% CI: 2.687-554.545, P=0.007), BTX-A injection (OR: 0.000; 95% CI, 0.000-0.078, P=0.004), TA injection (OR: 0.003; 95%CI: 0.000-0.452, P=0.023) and depth of infiltration (OR: 0.002; 95%CI: 0.000-0.359, P=0.020) were shown to be risk factors for the formation of esophageal strictures after ESD. As shown in Table 6, according to the OR value, BTX-A injection, TA injection, circumferential range and depth of infiltration were protective factors for the formation of esophageal strictures after ESD.

## Discussion

As the increasing numbers of ESD procedures performed, treatment of esophageal stricture is of great clinical importance to improve the quality of patients' life and decrease the burden of society 13. Previous studies have suggested that endoscopic injection of BTX-A or TA can prevent the occurrence of esophageal stenosis after ESD, when comparing with blank controls 10, 11. Our study is the first prospective clinical trial to confirm that compare with blank control, the endoscopic injection of BTX-A or TA significantly reduces the incidence of esophageal strictures after ESD treatment for early esophageal cancer. BTX-A or TA injection can also decrease the numbers of endoscopic bougie procedures required for stricture treatment. Additionally, in patients with entire circumference mucosal defect, the esophageal stricture rate was significantly lower in BTX-A injection than that in TA injection, which showed that BTX-A injection is superior to TA injection in larger circumference mucosal defect. Logistic regression analysis showed that BTX-A injection, TA injection and depth of infiltration were protective factors for the formation of esophageal strictures after ESD.

Steroids are commonly used for autoimmune and inflammatory diseases in the clinics 14. A meta-analysis indicated that long-term oral steroid appears to be the optimal prevention method for postoperative stricture formation compared with single-dose steroid injection, multiple-dose steroid injection, and steroid injection combined with oral steroid 15. Okamoto et al suggested that, endoscopic TA injection is not sufficient for preventing esophageal stricture in patients bearing mucosal defects covering more than seven-eighths of the esophageal circumference after ESD 16. According to our study, we found that single local TA injection or combined with oral prednisolone was not enough as prophylaxis for post-ESD strictures, especially for patients with complete circumference mucosal defect. Moreover, multiple-dose TA injection with repeated endoscopic procedures demands perfect operation and may increase the risk of perforation and hemorrhage 17. Besides, long treatment duration of oral steroid and its systemic effect may cause infection, worsen the condition of diabetes mellitus and increase the risk of osteoporosis 18.

It has been reported that the local application of BTX-A inhibited collagen deposition and fibrous connective tissue formation during the injury repair process in the skin, urethra, and joints 19. Also, BTX-A has been reported to reduce the movement of local muscles, decrease skin extension caused by muscle contraction, and limit the extent of inflammatory injury and tensile forces important in the process of scar formation 20. Numerous studies have suggested that intralesional BTX-A injections are useful for the treatment of keloids and hypertrophic scars 21, 22. Wen et al perform a randomized case-controlled trial and showed that single-dose BTX-A injection is highly effective in preventing esophageal strictures post-ESD, which was the first to apply the benefits of BTX-A injections on scar formation from the field of plastic surgery to the digestive system 11.

In this study, BTX-A was injected at the level of the muscularis propria, whereas TA was injected at the base of the artificial ulcer and must be injected into the submucosal layer. A potential problem with endoscopic TA injection is the risk of delayed perforation, which may occur if the steroid is injected into the true muscular layer 23. In order to achieve an effective administration of the steroid into the submucosal layer, operators were required to strip the lesions at the middle level of the submucosal layer to create enough space for the injection, which undoubtedly increased the difficulty of ESD procedure. Additionally, during the injection procedure, we found that the incidence of drug leakage was higher in TA injection than in BTX-A injection, which reduced the utilization ratio of TA 24.

There were several limitations of this study. First, the sample size of the study was small, and future randomized, double-blinded studies with larger sample sizes are needed. Second, the study was a single-center analysis and possible bias could not be eliminated.

## Conclusion

Endoscopic injection of BTX-A and TA was effective and safety in preventing post-ESD esophageal strictures and can decrease the times of bougie dilation. Particularly, BTX-A injection was more effective in patients with entire circumference mucosal defect, which is of great clinical importance to esophageal stricture patients after ESD procedures.

## Supplementary Material

Supplementary figures.

## Figures and Tables

**Table 1:** Baseline information of the three groups

	BTX-A (Group A) (n=26)	TA (Group B) (n=16)	Control (Group C) (n=36)	*p* value
Sex (male, n%)	65.4	62.5	72.2	0.741
Age (mean±SD, year)	65.15±7.23	65.06±7.88	65.28±8.11	0.995
Hospital stay (medium±Interquartile Range, day)	7±2.3	7±3	8±4	0.432
BMI (mean±SD)	23.35±2.72	23.47±3.04	23.83±3.03	
Smoking history (n%)	42.3	37.5	30.6	0.600
Drinking history (n%)	42.3	37.5	22.2	0.215
Family history of esophageal Tumors (n%)	15.4	6.3	16.7	0.701
Location of lesion				
Upper	2	1	1	0.553
Middle	13	10	25	
Lower	11	5	10	
Post-operative pathology				
HGIN/Carcinoma in situ	23	11	33	0.097
Squamous cell carcinoma	3	5	3	
Circumferential range				0.945
Two thirds to three fourths	9	4	9	
Three fourths to entire	11	8	17	
Full circumference	6	4	10	
Longitudinal resection length, mean±SD, cm	4.83±1.32	4.75±1.05	4.69±2.00	0.943
Depth of infiltration				0.129
Mucosal	23	11	24	
Submucosal	3	5	12	
ESD procedure time, min, median (range)	102.86±54.28	120.00±69.69	112.36±53.00	0.627
Rate of en-bloc resection	24/26	16/16	34/36	0.816
Adverse events				
Muscular injury	4	6	14	0.114
Hemorrhage	1	1	4	0.658

**Table 2:** Presentation of post-ESD esophageal stricture formation rates (per protocol set and intention to treat). Main Outcome Measurement: The incidence of esophageal strictures

	BTX-A (group A) (n=26)	TA (group B) (n=16)	Control (group C) (n=36)	*p* value
Proportion of patients developing stricture (intention to treat)	30.00% (9/30)	40.90% (9/22)	84.21% (32/38)	<0.001^*^
Proportion of patients developing stricture (per protocol)	26.92% (7/26)	43.75% (7/16)	83.33% (30/36)	<0.001^*^

*p<0.05

**Table 3:** Comparison of esophageal stricture incidence between each of the two groups

	BTX-A vs TA	BTX-A vs control	TA vs control
p	0.261	<0.001^*^	0.004^*^
χ2	1.262	19.964	8.456
OR (95%CI)	2.111 (0.567-7.855)	0.074 (0.021-0.253)	0.156 (0.042-0.583)

*p<0.05

**Table 4:** Numbers of patients experiencing esophageal stricture in the three groups

	BTX-A (group A) (n=26)	TA (group B) (n=16)	Control (Group C) (n=36)	Total stricture number
Two thirds to three fourths	0/9	0/4	5/9	5
Three fourths to entire	5/11	3/8	15/17	23
Full circumference	2/6	4/4	10/10	16
Total	7/26	7/16	30/36	44

*p<0.05

**Table 5:** Characteristics of the three groups after ESD procedure

	BTX-A (group A) (n=26)	TA (group B) (n=16)	Control (Group C) (n=36)	*P value*
Grading of dysphagia (range)	0.5 (0-3)	1.5 (0-3)	2 (0-4)	0.009^*^
Scores of EORTC QLQ-OES18, mean±SD	24.15±2.19	24.88±2.13	23.47±1.13	0.029^*^
No. of required bougie dilations, mean (range)	1.19 (0-12)	1.31 (0-9)	3.14 (0-16)	0.019^*^
Time that stricture occurred, mean±SD, days	43.5±37	40±20	32±50.5	0.643
Asymptomatic remission periods, mean±SD, days	46.6±10.6	49.5±8.8	26.4±6.5	0.001^*^
Diameter of narrow esophagus, mean±SD, mm	5.6±1.0	4.8±1.2	3.8±1.1	0.021^*^

*p<0.05

**Table 6:** Multivariate logistic regression analysis of the confounding factors in the development of postoperative esophageal strictures

Parameters	P value	Odds ratio	95% Confidence interval
Family history of esophageal tumor	0.537	2.200	0.179-26.968
Smoking history	0.654	0.605	0.067-5.437
Drinking history	0.597	1.873	0.183-19.162
ESD duration	0.277	1.013	0.990-1.037
Tumor location	0.197	0.302	0.049-1.862
post-operation pathology	0.638	3.746	0.015-917.086
Oral prednisolone	0.913	1.219	0.035-41.966
BTX-A injection	0.008^*^	0.000	0.000-0.120
TA injection	0.028^*^	0.002	0.000-0.514
Circumferential range	0.008^*^	25.192	2.289-227.263
Longitudinal resection length	0.693	0.847	0.371-1.933
Depth of infiltration	0.029^*^	0.001	0.000-0.507

*p<0.05

## Abbreviations

ESD: Endoscopic Submucosal Dissection
BTX-A: botulinum toxin type A
TA: triamcinolone acetonide
EMR: endoscopic mucosal resection
