# Supplementary material for: Comparison of endoscopic injection of botulinum toxin and steroids immediately after endoscopic submucosal dissection to prevent esophageal stricture: a prospective cohort study
Source: J Cancer. 2021 Aug 2;12(19):5789–96. doi: 10.7150/jca.60720 (PMC8408129; doi:10.7150/jca.60720)

Supplementary Figure 1: ESD procedures and postoperative gastroscope in patients receiving BTX-A injection. (A) Early esophageal cancer extending entire circumferential of the esophageal lumen. (B) Lugol's iodine solution was administered, and the lesion area was not stained. (C) Lesion was marked and the lift sign was positive. (D-E) The edge of the lesion was cut and separated step by step. (F) BTX-A was injected at muscularis propria by points. (G) The artificial ulcer caused by ESD, which resulted in a mucosal defect affecting entire circumference. (H) Follow-up endoscopy 6 weeks after ESD treatment revealed a white scar and no postoperative stricture.

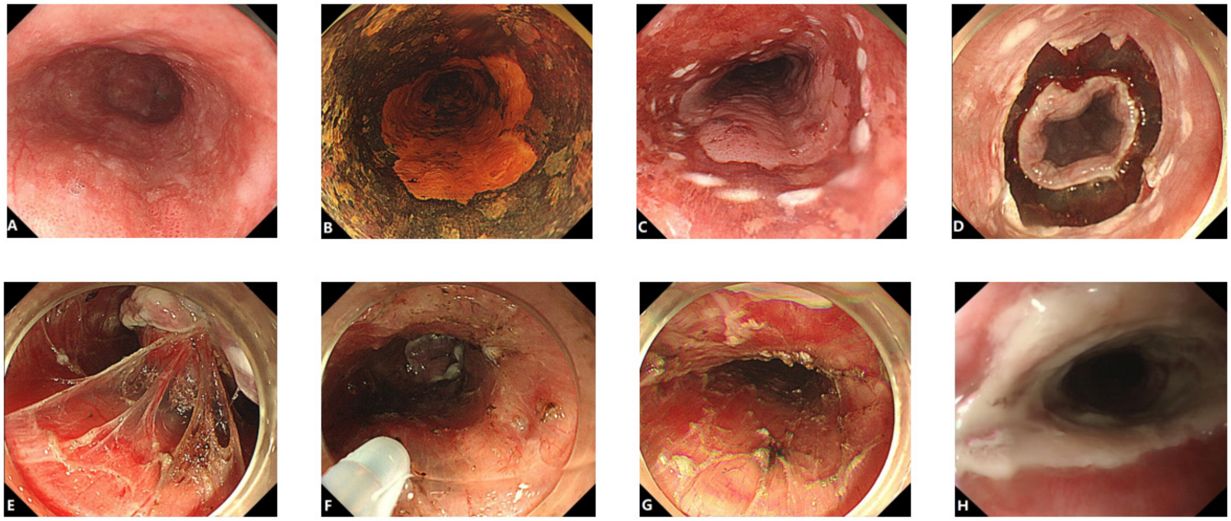

Supplementary Figure 2: ESD procedures and postoperative gastroscope in patients receiving TA injection. (A) Early esophageal cancer extending nearly four fifths of the circumference of the esophageal lumen. (B) Lugol's iodine solution was administered, and the lesion area was not stained. (C) Lesion was marked and the lift sign was positive. (D-E) The edge of the lesion was

cut and separated step by step. (F) The artificial ulcer caused by ESD, which resulted in a mucosal defect affecting more than four fifths of the circumference. (H) Follow-up endoscopy 6 weeks after ESD treatment revealed a white scar and no postoperative stricture.

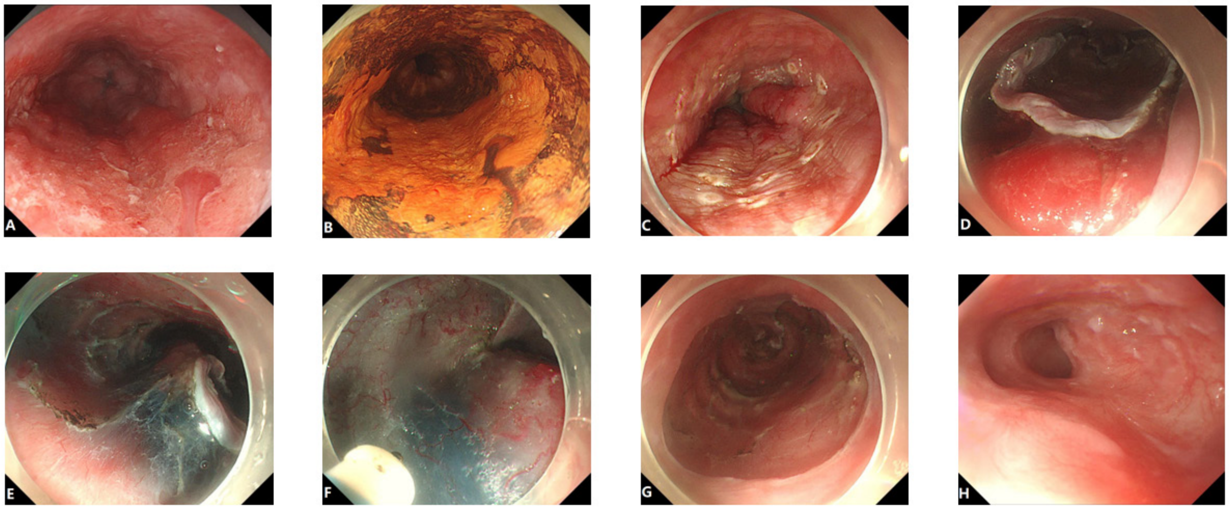

Supplement: Supplementary file 1 — Supplementary figures. [file jcav12p5789s1.pdf]
